# Impact of surgical aortic valve replacement and transcatheter aortic valve implantation on cardiovascular and cerebrovascular controls: A pilot study

**DOI:** 10.14814/phy2.70028

**Published:** 2024-09-03

**Authors:** Vlasta Bari, Francesca Gelpi, Beatrice Cairo, Martina Anguissola, Elena Acerbi, Mattia Squillace, Beatrice De Maria, Enrico Giuseppe Bertoldo, Valentina Fiolo, Edward Callus, Carlo De Vincentiis, Francesco Bedogni, Marco Ranucci, Alberto Porta

**Affiliations:** ^1^ Department of Biomedical Sciences for Health University of Milan Milan Italy; ^2^ Department of Cardiothoracic, Vascular Anesthesia and Intensive Care IRCCS Policlinico San Donato Milan Italy; ^3^ Department of Clinical and Interventional Cardiology IRCCS Policlinico San Donato Milan Italy; ^4^ IRCCS Istituti Clinici Scientifici Maugeri Milan Italy; ^5^ Clinical Psychology Service IRCCS Policlinico San Donato Milan Italy; ^6^ Department of Cardiac Surgery IRCCS Policlinico San Donato Milan Italy

**Keywords:** active standing, aortic valve stenosis, autonomic nervous system, baroreflex, blood flow, cerebral autoregulation, heart rate variability, mean arterial blood pressure

## Abstract

Surgical aortic valve replacement (SAVR) and transcatheter aortic valve implantation (TAVI) are options in severe aortic valve stenosis (AVS). Cardiovascular (CV) and cerebrovascular (CBV) control markers, derived from variability of heart period, systolic arterial pressure, mean cerebral blood velocity and mean arterial pressure, were acquired in 19 AVS patients (age: 76.8 ± 3.1 yrs, eight males) scheduled for SAVR and in 19 AVS patients (age: 79.9 + 6.5 yrs, 11 males) scheduled for TAVI before (PRE) and after intervention (POST, <7 days). Left ventricular function was preserved in both groups. Patients were studied at supine resting (REST) and during active standing (STAND). We found that: (i) both SAVR and TAVI groups featured a weak pre‐procedure CV control; (ii) TAVI ensured better CV control; (iii) cerebral autoregulation was working in PRE in both SAVR and TAVI groups; (iv) SAVR and TAVI had no impact on the CBV control; (v) regardless of group, CV and CBV control markers were not influenced by STAND in POST. Even though the post‐procedure preservation of both CV and CBV controls in TAVI group might lead to privilege this procedure in patients at higher risk, the missing response to STAND suggests that this advantage could be insignificant.

## 1 INTRODUCTION

Severe aortic valve stenosis (AVS) induces cardiac sympathetic nervous overactivation and impaired baroreflex function, thus affecting cardiovascular (CV) control (Dumonteil et al., 2013; Kadoya et al., 2020; Sobajima et al., 2018). The impact of AVS on cerebral circulation is less known. Since the increase of cardiac output after transcatheter aortic valve implantation (TAVI) is associated with an improvement of cerebral blood flow (CBF) (Vlastra et al., 2021), it can be hypothesized that AVS might lead to a chronic hypoperfusion of the brain (Caldas et al., 2017), even though this cerebrovascular (CBV) regulation deficit was not observed in all patients exhibiting a reduced ventricular ejection fraction (Caldas et al., 2019).

CV regulation can be evaluated from the analysis of the spontaneous fluctuations of the heart period (HP) (La Rovere et al., 2020; Task Force of the European Society of Cardiology and the North American Society of Pacing and Electrophysiology, 1996) and the analysis of the dynamic interactions between HP and systolic arterial pressure (SAP) (Laude et al., 2004; Porta et al., 2023). AVS is associated with a decrease of the magnitude of HP variability and its complexity (Valencia et al., 2009; Vukasovic et al., 1999), indicative of vagal withdrawal and sympathetic activation (Pomeranz et al., 1985; Porta et al., 2007), and with a lower baroreflex sensitivity (BRS), suggestive of baroreflex impairment (Bari et al., 2023).

CBV control can be evaluated from the analysis of the variability of mean CBF (MCBF), approximated via the mean cerebral blood velocity (MCBv) acquired via a transcranial Doppler device (Aaslid et al., 1982), and mean arterial pressure (MAP) (Panerai et al., 2022). AVS patients with preserved left ventricular function exhibited preserved cerebral autoregulation (CA) (Bari et al., 2023; Pedro et al., 2023; Porta et al., 2020; Porta, Gelpi, et al., 2022), even though cerebral vasoreactivity might be affected (Pedro et al., 2023).

AVS patients can receive indication for surgical aortic valve replacement (SAVR) or TAVI. TAVI is the preferred technique for patients at high surgical risk, but recent results indicated that TAVI might be applied even to patients at intermediate or low surgical risk (Ahmad et al., 2023; De Backer et al., 2016; Jørgensen et al., 2021; Mistiaen, 2023). Several studies assessed the impact of SAVR and TAVI on CV and CBV controls. It was observed that AVS patients featured a reduced respiratory sinus arrhythmia (RSA) and BRS after SAVR (Bari et al., 2023; Porta et al., 2020; Retzlaff et al., 2009; Retzlaff et al., 2011), while TAVI seems to preserve much better vagal control (Compostella et al., 2015; Retzlaff et al., 2011) and even to reduce sympathetic activity (Dumonteil et al., 2013). Fewer studies investigated the effect of SAVR and TAVI over the CBV control (Bari et al., 2023; Pedro et al., 2023; Porta et al., 2020; Porta, Gelpi, et al., 2022; Vlastra et al., 2021). Findings suggested that the CBV regulation is preserved after SAVR (Bari et al., 2023; Pedro et al., 2023; Porta et al., 2020; Porta, Gelpi, et al., 2022) and CBF is improved after TAVI because of the increased cardiac output (Vlastra et al., 2021). The assessment of the CV control is a relevant issue given that autonomic function and baroreflex control play a significant role in limiting the post‐procedure arrhythmic risk (Bari et al., 2018; Bauernschmitt et al., 2007) and episodes of orthostatic intolerance (Hanada et al., 2017). The evaluation of the CBV regulation is even more important given that neurological damage is frequently associated with SAVR and TAVI (Nogueira et al., 2021) with an incidence of ischemic stroke ranging from 2% to 6% after both SAVR and TAVI (Abdul‐Jawad Altisent et al., 2016; Kapadia et al., 2018), but increasing dramatically above 40% whether acute ischemic brain lesions are evaluated via brain diffusion‐weighted magnetic resonance imaging (Abdul‐Jawad Altisent et al., 2016) or silent stroke is considered (Grabert et al., 2016). Moreover, worsened CBV control has been associated with post‐procedure acute kidney dysfunction (Vaini et al., 2019).

Unfortunately, studies assessing the effects of TAVI and SAVR in age‐ and gender‐matched groups with preserved left ventricular function on CV and CBV controls are scarce. We hypothesize that SAVR and TAVI affect CV and CBV controls and these influences might be different. This information might be clinically relevant because it may concur in shaping the patient's post‐procedure risk profile, thus possibly influencing the decision about the most suitable procedure for treatment of AVS in patients with preserved left ventricular ejection fraction.

Thus, the aim of this study is to investigate CV and CBV controls in age‐ and gender‐matched groups scheduled for SAVR and TAVI with preserved left ventricular ejection fraction via the analysis of HP, SAP, MAP and MCBv spontaneous variability before (PRE) and after (POST) procedure. An orthostatic challenge, namely active standing (STAND), was utilized to probe the ability of CV and CBV regulatory mechanisms to respond to a physiological challenge (Bari et al., 2017; Cooke et al., 1999; Marchi et al., 2016a; Milan‐Mattos et al., 2018; Porta, Gelpi, et al., 2022). This challenge is useful even in elderly healthy subjects (Catai et al., 2014; De Maria et al., 2019; Gao et al., 2015; Laitinen et al., 1998; Laitinen et al., 2004; Milan‐Mattos et al., 2018).

## 2 MATERIALS AND METHODS

### 2.1 Experimental protocol

Nineteen severe AVS patients scheduled for SAVR (age: 76.8 ± 3.1 yrs, min‐max range: 73–83 yrs, eight males) and 19 severe AVS patients scheduled for TAVI (age: 79.9 + 6.5 yrs, min‐max range: 68–90 yrs, 11 males) were enrolled respectively at the Department of Cardiothoracic and Vascular Anesthesia and Intensive Care and at the Department of Clinical and Interventional Cardiology of IRCCS Policlinico San Donato, San Donato Milanese, Milan, Italy. The two groups were age‐ and gender‐matched. According to their medical records patients did not experience ischemic or hemorrhagic stroke or suffered for mental disorders before the procedure. All the patients were in sinus rhythm at the time of enrollment. The study was approved by San Raffaele Hospital ethical committee, Milan, Italy (approval number: 68/int/2018; approval date: 05/04/2018) and authorized by IRCCS Policlinico San Donato, San Donato Milanese, Milan, Italy (authorization date: 13/04/2018). Written signed informed consent was obtained from all the patients. SAVR patients underwent the standard general anesthesia protocol performed at Department of Cardiothoracic and Vascular Anesthesia and Intensive Care, IRCCS Policlinico San Donato. SAVR patients received a premedication with intramuscular atropine (0.5 mg) and fentanyl (100 μg) about 1 h before reaching the operating theater. Anesthesia was then induced with an intravenous bolus injection of propofol (1 mg·kg^−1^) and an infusion of remifentanil at 0.2 μg·kg^−1^·min^−1^. Maintenance of anesthesia was achieved with a continuous infusion of propofol at 2–3 mg·kg^−1^·h^−1^ and a remifentanil infusion ranging from 0.05 to 0.5 μg·kg^−1^·min^−1^. Additional inhalation agents (i.e., sevorane) could be used as requested. TAVI patients were treated under local anesthesia as to the standard of the Department of Clinical and Interventional Cardiology, IRCCS Policlinico San Donato at a dose of naropine (10 mL of 0.75% solution), plus an intravenous infusion at 0.03 μg·kg^−1^·min^−1^ of remifentanil and a bolus of 1–4 mg of midazolam and 50–100 μg of fentanyl.

We acquired electrocardiogram (ECG) from lead II via a bioamplifier (BioAmp FE132, ADInstruments, Australia), noninvasive arterial pressure (AP) from the middle finger via volume‐clamp photopletismography (CNAP Monitor 500, CNSystems, Austria) and cerebral blood velocity (CBv) via a transcranial Doppler device (Multi‐Dop X, DWL, San Juan Capistrano, CA, USA). The signals were analog‐to‐digital converted via a commercial device at a sampling rate of 400 Hz (PowerLab, ADinstruments, Australia). The subject was instrumented and left at rest in supine position (REST) for 10 min. Then, signals were recorded at REST and during STAND. Each session lasted 10 min with STAND session starting immediately after the REST one. The first 3 min of STAND were discarded when the signals were processed. Recording sessions took place before the procedure (PRE) and they were repeated within 7 days after the procedure (POST). CV control markers could be assessed in 19 SAVR patients in PRE both at REST and during STAND, in seven SAVR patients in POST both at REST and during STAND, in 17 TAVI patients in PRE both at REST and during STAND, and in nine TAVI patients in POST at REST and in eight TAVI patients in POST during STAND. As to the possibility of computing CV control markers, the relevant drop in SAVR individuals acquired in POST was justified by the postsurgical physical and psychological debilitation of the SAVR group and consequent refusal to participate to the POST session. The reduced possibility to assess CV control markers in TAVI subjects in PRE compared to the original size of the enrolled TAVI group was the result of poor signal quality in two subjects, while the decline in POST was the consequence of low signal quality in one subject and of the post‐intervention physical debilitation of the subject with consequent withdrawal from the protocol. CBV control markers were assessed in 10 SAVR patients in PRE at REST and in 11 at STAND, in six SAVR patients in POST at REST and in five at STAND, in nine TAVI patients in PRE at REST and in eight at STAND and in four TAVI patients in POST at REST and in three at STAND. As to the possibility of computing CBV control markers the relevant drop of the size in the SAVR and TAVI groups acquired in PRE compared to the original sizes of the enrolled groups was justified by the difficulty in insonating the middle cerebral arteries that it is known to become more and more important with age and pathology (Panerai et al., 2022). The additional drop of the size of both groups during STAND is the result of the post‐procedure physical debilitation. SAVR or TAVI patients who developed atrial fibrillation episodes or sustained ventricular arrhythmias during experimental sessions in POST were excluded from the POST group. The proportions of SAVR and TAVI patients who were excluded because they developed post‐procedure arrhythmic episodes were not significantly different between the two groups. No SAVR or TAVI patients experienced episodes of orthostatic hypertension/hypotension during STAND requiring the return to supine position. Consequently, at REST and during STAND recordings were free of sustained ventricular arrhythmias, spells of atrial fibrillation and evident AP instabilities associated with episodes of orthostatic hypertension/hypotension.

### 2.2 Variability series extraction and time domain CV and CBV control indexes

From the ECG, AP and CBv signals, beat‐to‐beat variability series of CV and CBV physiological variables were extracted. The HP was approximated as the time distance of two consecutive R‐wave peaks on the ECG located with minimum jitters through parabolic interpolation. SAP was defined as the maximum of the AP signal within the *n*th HP, while the *n*th diastolic value was the minimum of AP following the *n*th SAP. The diastolic values of CBv were taken as the minima of CBv signal temporally closest to the diastolic values. MAP and MCBv were computed as the integral between the time occurrences of (*n*–1) th and *n*th diastolic points detected on AP and CBv signals respectively divided by the interdiastolic interval (Bari et al., 2016). Values were checked manually by a trained operator and corrected in case of misdetections. The impact of arrhythmic cardiac beats was mitigated via linear interpolation between the closest reliable values. Stationary epochs of 256 consecutive values with stable mean and variance were extracted in all the subjects in any experimental condition (i.e., REST and STAND) and time point of analysis (i.e., PRE and POST). In the time domain we computed mean (μ) and variance (σ^2^) of HP, SAP, MAP and MCBv variability series, labeled as μ_HP_, σ^2^_HP_, μ_SAP_, σ^2^_SAP_, μ_MAP_, σ^2^_MAP_, μ_MCBv_, σ^2^_MCBv_ and expressed in ms, ms^2^, mmHg, mmHg^2^, mmHg, mmHg^2^, cm·s^−1^, and cm^2^·s^−2^ respectively. The values of σ^2^ were computed after subtraction of a linear trend.

### 2.3 Spectral and cross‐spectral CV and CBV control markers

After linear detrending, univariate spectral indexes of CV and CBV controls were calculated from power spectral density functions. Briefly, variability series were fitted with an autoregressive (AR) model (Baselli et al., 1997). The coefficients of the AR model were identified by solving the least squares problem via the Levinson‐Durbin recursion and their number was chosen according to the Akaike information criterion in the range between 8 and 14 (Akaike, 1974). Power spectral density was computed from the transfer function of the AR model and it was factorized into components according to the residual theorem (Baselli et al., 1997). Each spectral component was associated to a frequency band according to the value of its central frequency (Baselli et al., 1997). As to the CV regulation, a component was marked as low frequency (LF), or high frequency (HF), if its central frequency was in the range between 0.04 and 0.15 Hz and between 0.15 and 0.4 Hz respectively (Task Force of the European Society of Cardiology and the North American Society of Pacing and Electrophysiology, 1996). As to the CBV regulation, a component was labeled as very low frequency (VLF), LF or HF whether its central frequency was in the range between 0.02 and 0.07 Hz, between 0.07 and 0.15 Hz, and between 0.15 and 0.4 Hz respectively (Porta, Gelpi, et al., 2022). The superior limit of the LF band and the inferior limit of the HF band were adjusted with respect to (Panerai et al., 2022) to account for breathing rates slower than 0.2 Hz (Vaini et al., 2019). The power of HP variability series in the HF band (HF_HP_), expressed in ms^2^, is an estimate of the magnitude of the RSA and it was taken as an index of parasympathetic modulation directed to the sinus node (Pomeranz et al., 1985; Task Force of the European Society of Cardiology and the North American Society of Pacing and Electrophysiology, 1996), while the power of SAP variability series in the LF band (SAP_LF_), expressed in mmHg^2^, was taken as a marker of sympathetic modulation directed to the vasculature (Marchi et al., 2016b; Pagani et al., 1997). The power of MAP and MCBv variability series in the VLF, LF, and HF bands, labeled as VLF_MAP_, LF_MAP_, and HF_MAP_ and expressed in mmHg^2^, and labeled as VLF_MCBv_, LF_MCBv_, and HF_MCBv_ and expressed in cm^2^·s^−2^, was calculated to assess the CBV control.

After linear detrending, bivariate cross‐spectral indexes of CV and CBV controls were calculated from power cross‐spectral density function (Panerai et al., 2022). Cross‐spectral indexes were calculated according to the bivariate AR approach (Porta, Gelpi, et al., 2022). The model parameters were identified by solving the least squares problem via the Cholesky decomposition method (Porta et al., 2000). The model order was fixed to 10 (Porta et al., 2000). Cross‐spectral and spectral density functions were computed from the coefficients of the model. Transfer function gain (TFG) was estimated as the ratio of the modulus of the cross‐spectral density function from the input to the output to the power spectral density of the input. Squared coherence (K^2^) was estimated as the ratio of the square modulus of the cross‐spectral density function from the input to the output to the product of the power spectral densities of the input and the output. K^2^ always ranged between 0 and 1, where 0 means null coupling and 1 indicates full coupling between input and output. In the case of the CV control analysis the input was SAP and the output was HP, thus characterizing the cardiac arm of the baroreflex (Laude et al., 2004), while in the case of the CBV control analysis the input was MAP and the output was MCBv, thus characterizing the dynamic component of the CA (Panerai et al., 2022). TFG and K^2^ were sampled in correspondence of the maximum of the K^2^ function in the considered frequency band of the CV and CBV controls (Bari et al., 2019). TFG from SAP to HP (TFG_HP‐SAP_) was expressed in ms·mmHg^−1^ and TFG from MAP to MCBv (TFG_MCBv‐MAP_) was expressed in cm·s^−1^·mmHg^−1^. TFG_HP‐SAP_ markers were labeled as TFG_HP‐SAP_(LF) and TFG_HP‐SAP_(HF), while TFG_MCBv‐MAP_ markers were denoted as TFG_MCBv‐MAP_(VLF), TFG_MCBv‐MAP_(LF), and TFG_MCBv‐MAP_(HF). K^2^ between HP and SAP (K^2^_HP,SAP_) and K^2^ between MCBv and MAP (K^2^_MCBv,MAP_) were dimensionless. K^2^_HP,SAP_ markers were labeled as K^2^_HP,SAP_(LF) and K^2^_HP,SAP_(HF), while K^2^_MCBv,MAP_ markers were denoted as K^2^_MCBv,MAP_(VLF), K^2^_MCBv,MAP_(LF), and K^2^_MCBv,MAP_(HF). The autoregulation index (ARI) was assessed via a time domain technique feeding the Tiecks' model (Tiecks et al., 1995) with MAP variability and measuring the discrepancy between predicted and measured MCBv variability series using the normalized mean square prediction error. The CA efficiency was graded using the categories provided in (Tiecks et al., 1995) and the approach selected the one that led to the minimal normalized mean square prediction error (Gelpi et al., 2022; Mahdi et al., 2017). ARI ranges from 0 to 9 with 4 being the limit between impaired (≤4) and preserved (>4) CA. The percentage of subjects with ARI >4 (%ARI >4) was computed as well.

### 2.4 Statistical analysis

Clinical and demographical data of the SAVR and TAVI groups were compared via t test, or Mann–Whitney rank sum test when appropriate, in the case of continuous variables, and chi‐square test, in case of categorical variables. Given the lack of data about the comparison of CV and CBV markers in age‐matched SAVR and TAVI populations, the size of the groups was selected according to our previous experience on the analysis of the SAVR population (Bari et al., 2023; Porta et al., 2020) suggesting that the impact of the surgical procedure on the CV control could be detectable via the variance of HP with less than 20 subjects even when accounting for the number of comparisons present in this study. After pooling the data relevant to REST and STAND, two‐way analysis of variance (Holm–Sidak test to deal with multiple comparison issue) was used to check the significance of the differences between the groups (i.e., SAVR and TAVI) within the same time point of analysis (i.e., PRE and POST) and between experimental time points within the same group. If normality test (Shapiro–Wilk test) and equal variance test (Brown‐Forsythe test) for the application of Holm‐Sidak test were not passed, the Mann‐Whitey rank sum test was applied. This test was substituted with Wilcoxon signed rank test when possible. The level of significance of each Mann‐Whitey rank sum test, or Wilcoxon signed rank test, was divided by the number of comparisons (i.e., 4) to account for the multiple comparison issue. The same statistical tests were applied in PRE and POST to check the significance of the differences between the groups (i.e., SAVR and TAVI) within the same experimental condition (i.e., REST or STAND) and between experimental conditions within the same group. Analyses were carried out over CV and CBV time and frequency domain markers. Chi‐square test was utilized to assess the significance of the modifications of %ARI >4 across groups and experimental conditions and, after pooling data relevant to REST and STAND, across groups and time points. Again, the level of significance of each Chi‐square test was divided by the number of comparisons (i.e., 4) to account for the multiple comparison issue. A commercial statistical software (Sigmaplot 14.5, Systat, Inc., Chicago, US) was utilized. The level of significance was set to 0.05. A type I error probability *p* below the level of significance, eventually corrected by the number of comparisons, was always deemed as significant.

## 3 RESULTS

Table 1 summarizes demographical and clinical data of SAVR and TAVI patients. No difference was present between clinical and demographic markers with the sole exception of the longer hospital stay in SAVR patients compared to TAVI subjects. Values of perioperative arterial partial pressure of carbon dioxide, as measured just before starting the anesthesiologic procedure from the radial artery, were not significantly different. PRE sessions took place 1.79 ± 1.75 days before SAVR and 1.42 ± 0.69 days before TAVI, while the POST ones 5.75 ± 1.04 days after SAVR and 3.78 ± 2.17 days after TAVI. The proportion of the patients under a specific pharmacological treatment did not vary in POST compared to PRE. None of the patients experienced fall episodes during their hospital stay.

**TABLE 1:** Clinical and demographic data of SAVR and TAVI patients.

Parameter	SAVR (*n* = 19)	TAVI (*n* = 19)
Age [yrs]	76.4 ± 2.9	79.9 ± 6.5
Gender [male]	8 (42)	11 (58)
BMI [kg·m^−2^]	26.5 ± 4.2	28.7 ± 6.0
AVS	19 (100)	19 (100)
Congestive heart failure	0 (0)	0 (0)
Recent myocardial infarction	0 (0)	0 (0)
Previous cerebrovascular events	1 (5)	1 (5)
History of recurrent falls	0 (0)	0 (0)
LVEF [%]	60.9 ± 9.8	58.8 ± 9.4
Perioperative pco_2_ [mmhg]	35.8 ± 4.8	38.0 ± 6.7
COPD	1 (5)	2 (11)
Serum creatinine [mg·dl^−1^]	0.95 ± 0.34	0.95 ± 0.35
Bilirubin [mg·dl^−1^]	0.82 ± 0.39	0.71 ± 0.24
Hypertension	8 (42)	15 (79)
HTC [%]	39.3 ± 4.9	39.5 ± 10.6
ACEF score	1.28 ± 0.39	1.42 ± 0.34
ACE inhibitors	8 (42)	6 (32)
STS score [%]	2.11 ± 0.98	2.43 ± 1.06
Beta‐blockers	10 (53)	3 (21)
Calcium antagonists	3 (16)	4 (21)
Hospital stay [days]	8.0 ± 4.2	4.3 ± 1.6§
Postoperative overt stroke	0 (0)	0 (0)
Postoperative atrial fibrillation	8 (42)	2 (11)
Postoperative acute complications	1 (5)	4 (21)
Hospital death	1 (5)	0 (0)

*Note:* Continuous data are presented as mean ± standard deviation and categorical data as number (percentage). The symbol § indicates *p* < 0.05 versus SAVR.

Abbreviations: ACE, angiotensin converting enzyme; ACEF, age creatinine ejection fraction; AVS, aortic valve stenosis; BMI = body mass index; COPD, chronic obstructive pulmonary disease; HTC, hematocrit; LVEF, left ventricular ejection fraction; pCO_2_: arterial partial pressure of carbon dioxide; STS, Society of Thoracic Surgeons.

Table 2 lists CV control markers in SAVR and TAVI patients extracted in PRE at REST and during STAND. No differences were visible across populations and experimental conditions with the notable exception of σ^2^_SAP_ that increased during STAND compared to REST in SAVR group becoming during STAND larger in SAVR patients than in TAVI individuals.

**TABLE 2:** CV control markers in SAVR and TAVI patients in PRE at REST and during STAND.

Marker	SAVR (*n* = 19)		TAVI (*n* = 19)	
	REST (*n* = 19)	STAND (*n* = 19)	REST (*n* = 17)	STAND (*n* = 17)
μ_HP_ [ms]	907 ± 123	832 ± 120	904 ± 135	860 ± 150
σ^2^_HP_ [ms^2^]	663 ± 743	517 ± 447	593 ± 699	474 ± 739
HF_HP_ [ms^2^]	95 ± 154	91 ± 161	210 ± 421	191 ± 523
μ_SAP_ [mmHg]	149 ± 20	139 ± 24	145 ± 20	140 ± 30
σ^2^_SAP_ [mmHg^2^]	25 ± 19	39 ± 34#	21 ± 14	24 ± 13§
LF_SAP_ [mmHg^2^]	2.2 ± 3.5	3.9 ± 4.3	2.0 ± 2.5	4.4 ± 4.7
TFG_HP‐SAP_(LF) [ms∙mmHg^−1^]	4.0 ± 3.4	2.7 ± 2.5	3.4 ± 3.3	2.2 ± 1.1
K^2^_HP,SAP_(LF)	0.37 ± 0.22	0.40 ± 0.18	0.35 ± 0.15	0.35 ± 0.22
TFG_HP‐SAP_(HF) [ms∙mmHg^−1^]	5.0 ± 6.1	4.3 ± 5.2	6.3 ± 6.0	2.9 ± 1.7
K^2^_HP,SAP_(HF)	0.57 ± 0.24	0.50 ± 0.24	0.55 ± 0.23	0.42 ± 0.20

*Note:* Results are reported as mean ± standard deviation. The symbol # indicates *p* < 0.05 versus REST within the same group, while the symbol § indicates *p* < 0.05 versus SAVR within the same experimental condition.

Abbreviations: HF, high frequency; HF_HP_, power of the HP series in the HF band; HP, heart period; K^2^, squared coherence; K^2^_HP,SAP_, K^2^ between HP and SAP series; K^2^_HP,SAP_(HF), K^2^_HP,SAP_ in the HF band; K^2^_HP,SAP_(LF), K^2^_HP,SAP_ in the LF band; LF, low frequency; LF_SAP_, power of the SAP series in the LF band; SAP, systolic arterial pressure; TFG, transfer function gain; TFG_HP‐SAP_, TFG from SAP to HP; TFG_HP‐SAP_(HF), TFG_HP‐SAP_ in the HF band; TFG_HP‐SAP_(LF), TFG_HP‐SAP_ in the LF band; μ_HP_, HP mean; μ_SAP_, SAP mean; σ^2^_HP_, HP variance; σ^2^_SAP_, SAP variance.

Table 3 summarizes CV control markers in SAVR and TAVI patients extracted in POST at REST and during STAND. CV variability indexes did not vary with either experimental condition or group.

**TABLE 3:** CV control markers in SAVR and TAVI patients in POST at REST and during STAND.

Marker	SAVR (n = 19)		TAVI (n = 19)	
	REST (*n* = 7)	STAND (n = 7)	REST (*n* = 9)	STAND (*n* = 8)
μ_HP_ [ms]	755 ± 111	725 ± 126	870 ± 176	762 ± 195
σ^2^_HP_ [ms^2^]	56 ± 50	315 ± 658	347 ± 473	393 ± 601
HF_HP_ [ms^2^]	24 ± 41	123 ± 279	62 ± 63	117 ± 252
μ_SAP_ [mmHg]	132 ± 17	140 ± 22	127 ± 15	140 ± 22
σ^2^_SAP_ [mmHg^2^]	20 ± 18	28 ± 20	27 ± 19	49 ± 37
LF_SAP_ [mmHg^2^]	1.0 ± 1.9	3.6 ± 2.4	6.0 ± 7.5	4.6 ± 5.5
TFG_HP‐SAP_(LF) [ms∙mmHg^−1^]	1.1 ± 0.8	0.9 ± 1.4	1.6 ± 1.1	1.2 ± 1.1
K^2^_HP,SAP_(LF)	0.27 ± 0.16	0.20 ± 0.10	0.39 ± 0.22	0.28 ± 0.18
TFG_HP‐SAP_(HF) [ms∙mmHg^−1^]	1.2 ± 1.1	4.1 ± 5.5	3.5 ± 4.4	3.2 ± 3.4
K^2^_HP,SAP_(HF)	0.63 ± 0.29	0.56 ± 0.19	0.42 ± 0.24	0.44 ± 0.21

*Note:* Results are reported as mean ± standard deviation.

Abbreviations: HF, high frequency; HF_HP_, power of the HP series in the HF band; HP, heart period; K^2^, squared coherence; K^2^_HP,SAP_, K^2^ between HP and SAP series; K^2^_HP,SAP_(HF), K^2^_HP,SAP_ in the HF band; K^2^_HP,SAP_(LF), K^2^_HP,SAP_ in the LF band; LF, low frequency; LF_SAP_, power of the SAP series in the LF band; SAP, systolic arterial pressure; TFG, transfer function gain; TFG_HP‐SAP_, TFG from SAP to HP; TFG_HP‐SAP_(HF), TFG_HP‐SAP_ in the HF band; TFG_HP‐SAP_(LF), TFG_HP‐SAP_ in the LF band; μ_HP_, HP mean; μ_SAP_, SAP mean; σ^2^_HP_, HP variance; σ^2^_SAP_, SAP variance.

Table 4 lists CBV control markers in SAVR and TAVI patients extracted in PRE at REST and during STAND. No differences were visible across populations and experimental conditions.

**TABLE 4:** CBV control markers in SAVR and TAVI patients in PRE at REST and during STAND.

Marker	SAVR (n = 19)		TAVI (n = 19)	
	REST (*n* = 10)	STAND (*n* = 11)	REST (n = 9)	STAND (n = 8)
μ_MCBv_ [cm∙s^−1^]	50.7 ± 23.6	49.8 ± 24.1	49.4 ± 20.8	51.7 ± 26.0
σ^2^_MCBv_ [cm^2^∙s^−2^]	33.2 ± 72.2	48.55 ± 110.1	21.7 ± 26.0	51.1 ± 62.7
VLF_MCBv_ [cm^2^∙s^−2^]	3.8 ± 5.1	2.6 ± 3.6	3.5 ± 4.4	9.6 ± 15.4
LF_MCBv_ [cm^2^∙s^−2^]	2.2 ± 4.6	3.3 ± 4.7	1.4 ± 1.7	7.8 ± 15.0
HF_MCBv_ [cm^2^∙s^−2^]	7.6 ± 16.1	13.2 ± 31.3	6.3 ± 8.9	8.8 ± 10.4
μ_MAP_ [mmHg]	98.4 ± 10.9	93.1 ± 17.0	104.5 ± 20.0	95.4 ± 22.3
σ^2^_MAP_ [mmHg^2^]	12.4 ± 6.9	22.2 ± 19.6	20.1 ± 14.1	19.8 ± 13.1
VLF_MAP_ [mmHg^2^]	2.8 ± 4.6	4.5 ± 7.5	7.4 ± 9.9	6.4 ± 12.7
LF_MAP_ [mmHg^2^]	1.9 ± 1.6	5.1 ± 9.7	2.0 ± 3.2	2.8 ± 3.6
HF_MAP_ [mmHg^2^]	2.2 ± 1.3	3.7 ± 3.9	4.5 ± 3.4	3.6 ± 2.4
TFG_MCBv‐MAP_(VLF) [cm∙s^−1^∙mmHg^−1^]	0.58 ± 0.34	0.56 ± 0.30	0.48 ± 0.30	0.43 ± 0.22
K^2^_MCBv,MAP_(VLF)	0.33 ± 0.23	0.37 ± 0.23	0.38 ± 0.29	0.31 ± 0.28
TFG_MCBv‐MAP_(LF) [cm∙s^−1^∙mmHg^−1^]	0.49 ± 0.27	0.67 ± 0.40	0.55 ± 0.38	0.45 ± 0.32
K^2^_MCBv,MAP_(LF)	0.29 ± 0.21	0.39 ± 0.23	0.22 ± 0.15	0.27 ± 0.23
TFG_MCBv‐MAP_(HF) [cm∙s^−1^∙mmHg^−1^]	0.98 ± 1.12	0.89 ± 0.49	0.80 ± 0.74	0.76 ± 0.48
K^2^_MCBv,MAP_(HF)	0.42 ± 0.26	0.41 ± 0.22	0.51 ± 0.25	0.39 ± 0.19
ARI	6.7 ± 3.1	7.7 ± 1.8	5.4 ± 3.5	5.6 ± 3.2
%ARI >4	80	91	67	75

*Note:* Results are reported as mean ± standard deviation.

Abbreviations: %ARI >4, percentage of subjects with ARI >4; ARI, cerebral autoregulation index; HF, high frequency; HF_MAP_, power of the MAP series in the HF band; HF_MCBv_, power of the MCBv series in the HF band; K^2^, squared coherence; K^2^_MCBv,MAP_, K^2^ between MCBv and MAP series; K^2^_MCBv,MAP_(HF), K^2^_MCBv,MAP_ in the HF band; K^2^_MCBv,MAP_(LF), K^2^_MCBv,MAP_ in the LF band; K^2^_MCBv,MAP_(VLF), K^2^_MCBv,MAP_ in the VLF band; LF = low frequency; LF_MAP_, power of the MAP series in the LF band; LF_MCBv_, power of the MCBv series in the LF band; MAP, mean arterial pressure; MCBv, mean cerebral blood velocity; TFG, transfer function gain; TFG_MCBv‐MAP_, TFG from MAP to MCBv; TFG_MCBv‐MAP_(HF), TFG_MCBv‐MAP_ in the HF band; TFG_MCBv‐MAP_(LF), TFG_MCBv‐MAP_ in the LF band; TFG_MCBv‐MAP_(VLF), TFG_MCBv‐MAP_ in the VLF band; VLF, very low frequency; VLF_MAP_, power of the MAP series in the VLF band; VLF_MCBv_, power of the MCBv series in the VLF band; μ_MAP_, MAP mean; μ_MCBv_, MCBv mean; σ^2^_MAP_, MAP variance; σ^2^_MCBv_, MCBv variance.

Table 5 summarizes CBV control markers in SAVR and TAVI patients extracted in POST at REST and during STAND. CBV variability indexes remained stable across either experimental conditions or groups with the notable exceptions of σ^2^_MAP_, VLF_MAP_, and TFG_MCBv‐MAP_(HF): σ^2^_MAP_ increased during STAND compared to REST in SAVR group, becoming during STAND larger in SAVR patients than in TAVI individuals, VLF_MAP_ was larger in TAVI group than in the SAVR one at REST, and TFG_MCBv‐MAP_(HF) was smaller during STAND compared to REST in the TAVI group.

**TABLE 5:** CBV control markers in SAVR and TAVI patients in POST at REST and during STAND.

Marker	SAVR (*n* = 19)		TAVI (*n* = 19)	
	REST (*n* = 6)	STAND (*n* = 5)	REST (*n* = 4)	STAND (*n* = 3)
μ_MCBv_ [cm∙s^−1^]	45.1 ± 13.9	32.7 ± 6.9	47.9 ± 19.8	43.4 ± 28.7
σ^2^_MCBv_ [cm^2^∙s^−2^]	12.9 ± 5.5	20.9 ± 17.8	11.5 ± 8.4	12.6 ± 18.7
VLF_MCBv_ [cm^2^∙s^−2^]	5.0 ± 6.3	10.3 ± 16.2	4.1 ± 4.8	0.3 ± 0.5
LF_MCBv_ [cm^2^∙s^−2^]	1.1 ± 1.4	1.7 ± 1.2	1.5 ± 2.1	1.4 ± 2.1
HF_MCBv_ [cm^2^∙s^−2^]	2.4 ± 1.4	2.3 ± 1.6	3.3 ± 3.9	2.0 ± 2.3
μ_MAP_ [mmHg]	87.9 ± 22.1	106.6 ± 27.4	92.9 ± 19.3	96.4 ± 13.1
σ^2^_MAP_ [mmHg^2^]	9.1 ± 2.2	15.1 ± 3.7#	9.7 ± 4.3	9.4 ± 5.1§
VLF_MAP_ [mmHg^2^]	0.5 ± 0.8	2.9 ± 4.0	5.0 ± 2.9§	1.9 ± 1.7
LF_MAP_ [mmHg^2^]	1.1 ± 1.0	1.7 ± 1.7	1.0 ± 0.7	1.7 ± 1.4
HF_MAP_ [mmHg^2^]	3.6 ± 2.9	3.8 ± 3.0	1.4 ± 0.9	1.4 ± 0.4
TFG_MCBv‐MAP_(VLF) [cm∙s^−1^∙mmHg^−1^]	0.68 ± 0.94	1.05 ± 1.14	1.10 ± 0.54	0.05 ± 2.46
K^2^_MCBv,MAP_(VLF)	0.35 ± 0.26	0.15 ± 0.09	0.49 ± 0.36	0.15 ± 0.08
TFG_MCBv‐MAP_(LF) [cm∙s^−1^∙mmHg^−1^]	1.19 ± 0.88	0.88 ± 1.07	1.19 ± 1.31	1.19 ± 1.76
K^2^_MCBv,MAP_(LF)	0.26 ± 0.25	0.17 ± 0.12	0.33 ± 0.25	0.13 ± 0.03
TFG_MCBv‐MAP_(HF) [cm∙s^−1^∙mmHg^−1^]	0.57 ± 0.10	0.52 ± 0.30	0.84 ± 0.36	0.29 ± 0.28#
K^2^_MCBv,MAP_(HF)	0.47 ± 0.33	0.27 ± 0.17	0.51 ± 0.33	0.17 ± 0.02
ARI	5.7 ± 3.1	6.2 ± 2.3	7.7 ± 1.3	7.0 ± 1.7
%ARI >4	67	80	100	100

*Note:* Results are reported as mean ± standard deviation. The symbol # indicates *p* < 0.05 versus REST within the same group, while the symbol § indicates *p* < 0.05 versus SAVR within the same experimental condition.

Abbreviations: %ARI >4, percentage of subjects with ARI >4; ARI, cerebral autoregulation index; HF, high frequency; HF_MAP_, power of the MAP series in the HF band; HF_MCBv_, power of the MCBv series in the HF band; K^2^, squared coherence; K^2^_MCBv,MAP_, K^2^ between MCBv and MAP series; K^2^_MCBv,MAP_(HF), K^2^_MCBv,MAP_ in the HF band; K^2^_MCBv,MAP_(LF), K^2^_MCBv,MAP_ in the LF band; K^2^_MCBv,MAP_(VLF), K^2^_MCBv,MAP_ in the VLF band; LF = low frequency; LF_MAP_, power of the MAP series in the LF band; LF_MCBv_, power of the MCBv series in the LF band; MAP, mean arterial pressure; MCBv, mean cerebral blood velocity; TFG, transfer function gain; TFG_MCBv‐MAP_, TFG from MAP to MCBv; TFG_MCBv‐MAP_(HF), TFG_MCBv‐MAP_ in the HF band; TFG_MCBv‐MAP_(LF), TFG_MCBv‐MAP_ in the LF band; TFG_MCBv‐MAP_(VLF), TFG_MCBv‐MAP_ in the VLF band; VLF, very low frequency; VLF_MAP_, power of the MAP series in the VLF band; VLF_MCBv_, power of the MCBv series in the VLF band; μ_MAP_, MAP mean; μ_MCBv_, MCBv mean; σ^2^_MAP_, MAP variance; σ^2^_MCBv_, MCBv variance.

**FIGURE 1:**
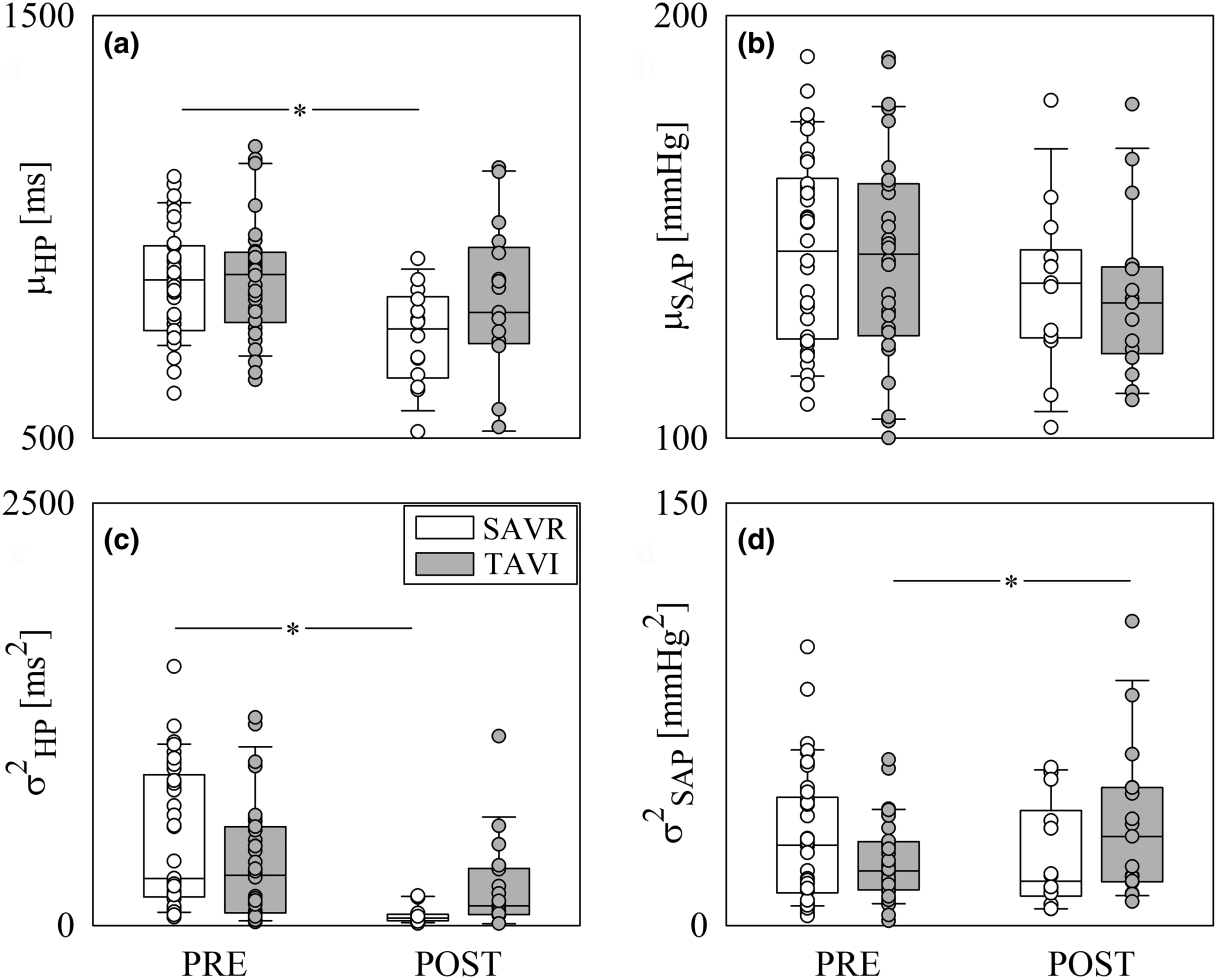
The vertical grouped box‐and‐whisker plots show μ_HP_ (a), μ_SAP_ (b), σ^2^_HP_ (c) and σ^2^_SAP_ (d) as a function of the time point of the recording (i.e., PRE and POST) in SAVR (white bars) and TAVI (gray bars) patients. Data are pooled together regardless the experimental condition (i.e., REST and STAND). The height of the box represents the distance between the first and third quartiles, with the median marked as a line, and the whiskers show the 5th and 95th percentiles. Individual values are reported as circles. The symbol * indicates *p* < 0.05 between different time points within the same population.

The vertical grouped box‐and‐whisker plots of the Figure 1 show time domain CV markers, namely μ_HP_ (Figure 1a), μ_SAP_ (Figure 1b), σ^2^_HP_ (Figure 1c) and σ^2^_SAP_ (Figure 1d) as a function of the time point of the analysis (i.e., PRE and POST) in SAVR (white bars) and TAVI (gray bars) populations. Data were pooled together regardless the experimental condition (i.e., REST or STAND). The height of the box represents the distance between the first and third quartiles, with the median marked as a line, and the whiskers show the 5th and 95th percentiles. Individual values were shown as circles. μ_HP_ and σ^2^_HP_ deceased in POST with respect to PRE only in the SAVR group (Figure 1a,c). μ_SAP_ did not vary with the time point of the analysis regardless of the group (Figure 1b), while σ^2^_SAP_ increased during POST compared to PRE only in the TAVI group. None of the time domain CV markers was able to differentiate groups and this conclusion held regardless of the time point of the analysis (Figure 1a–d). The layout of Figure 1 was exploited for all the remaining figures.

**FIGURE 2:**
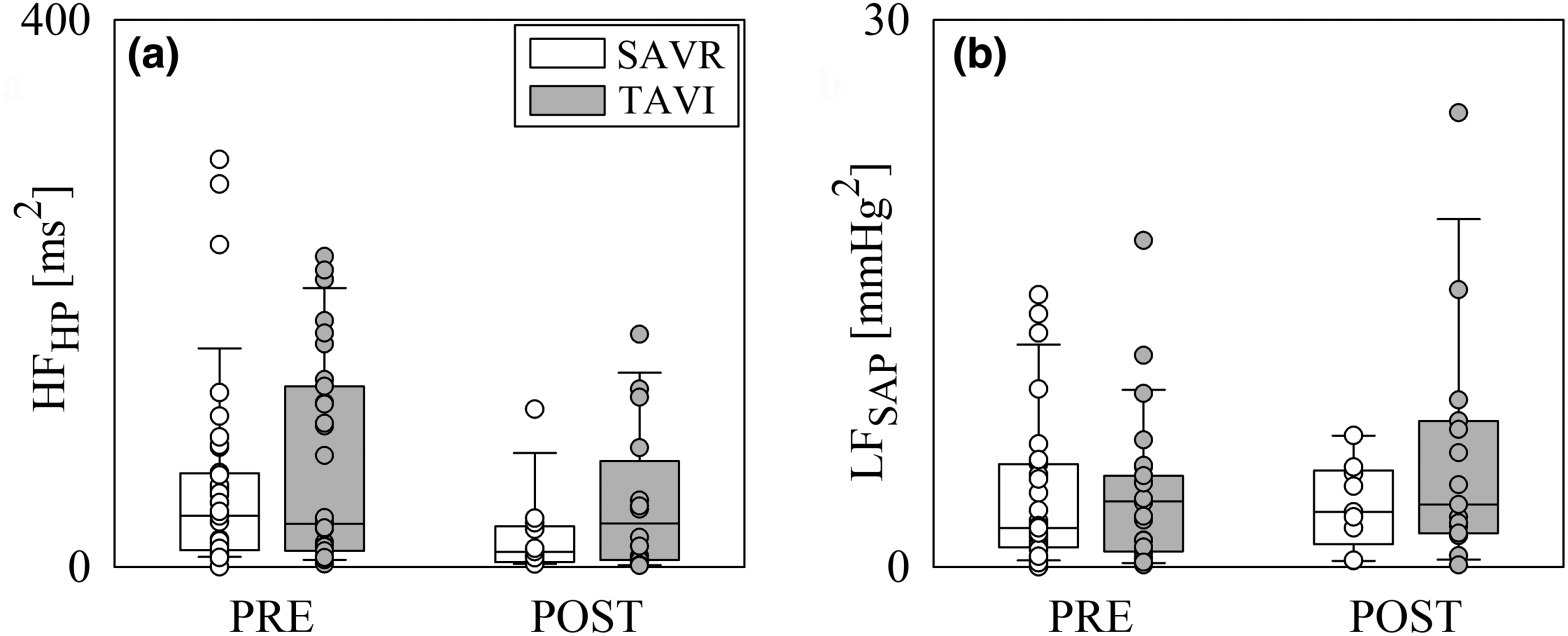
The vertical grouped box‐and‐whisker plots show HF_HP_ (a) and LF_SAP_ (b) as a function of the time point of the recording (i.e., PRE and POST) in SAVR (white bars) and TAVI (gray bars) patients. Data are pooled together regardless the experimental condition (i.e., REST and STAND). The height of the box represents the distance between the first and third quartiles, with the median marked as a line, and the whiskers show the 5th and 95th percentiles. Individual values are reported as circles.

Figure 2 shows HF_HP_ (Figure 2a) and LF_SAP_ (Figure 2b) powers. HF_HP_ and LF_SAP_ powers did not change across either populations or time points (Figure 2a,b).

**FIGURE 3:**
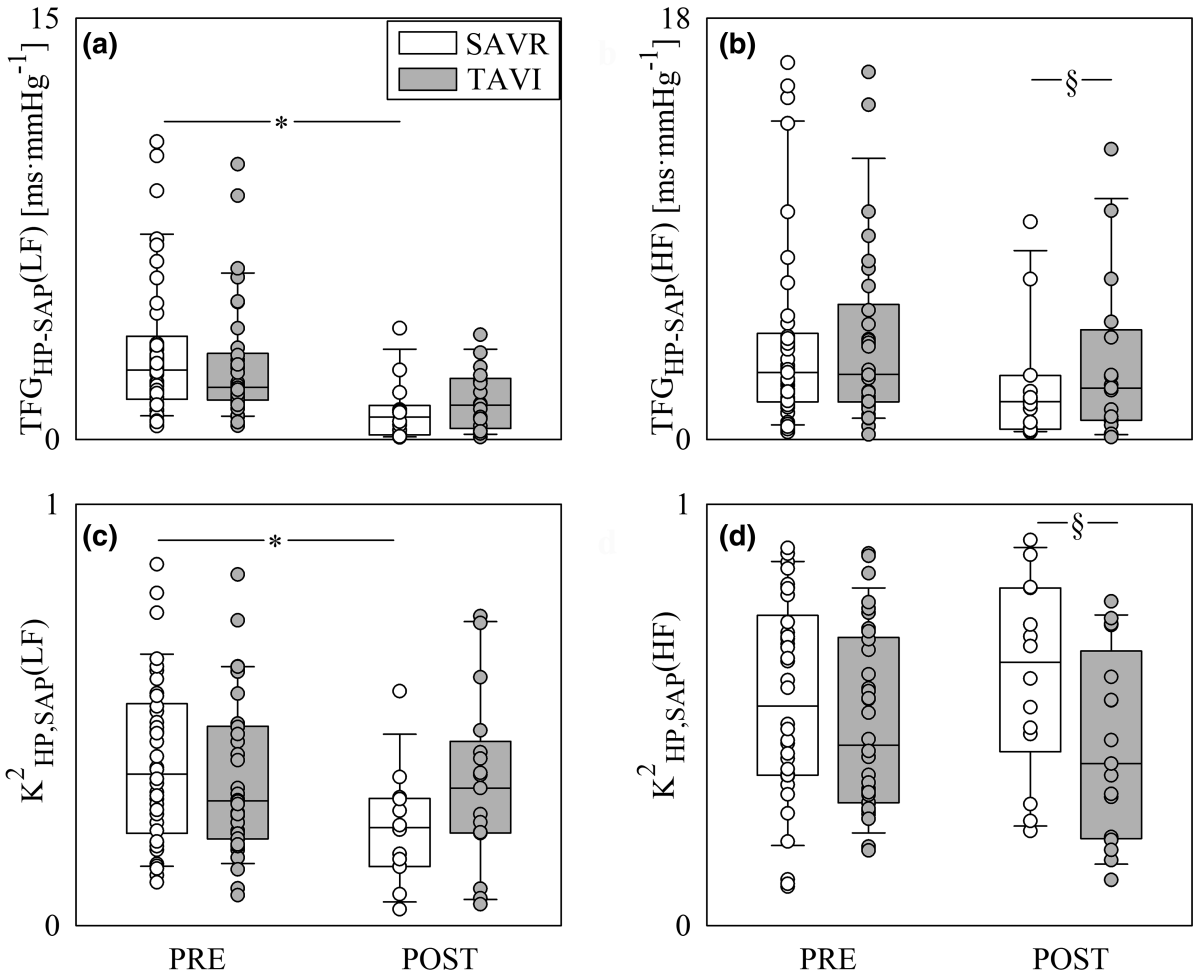
The vertical grouped box‐and‐whisker plots show TFG_HP‐SAP_(LF) (a), TFG_HP‐SAP_(HF) (b), K^2^_HP,SAP_(LF) (c) and K^2^_HP,SAP_(HF) (d) as a function of the time point of the recording (i.e., PRE and POST) in SAVR (white bars) and TAVI (gray bars) patients. Data are pooled together regardless the experimental condition (i.e., REST and STAND). The height of the box represents the distance between the first and third quartiles, with the median marked as a line, and the whiskers show the 5th and 95th percentiles. Individual values are reported as circles. The symbol * indicates *p* < 0.05 between different time points within the same population, while the symbol § indicates *p* < 0.05 between different populations within the same time point of recording.

Figure 3 shows TFG_HP‐SAP_(LF) (Figure 3a), TFG_HP‐SAP_(HF) (Figure 3b), K^2^_HP,SAP_(LF) (Figure 3c), and K^2^_HP,SAP_(HF) (Figure 3d). TFG_HP‐SAP_ tended to decrease in POST compared to PRE and this tendency held regardless of frequency band and groups (Figure 3a,b). However, this decrease was significant only in the case of TFG_HP‐SAP_(LF) in the SAVR population (Figure 3a). TFG_HP‐SAP_ indexes were not significantly different between SAVR and TAVI groups in both PRE and POST (Figure 3a,b), with the notable exception of TFG_HP‐SAP_(HF), being in POST significantly larger in the TAVI group than in the SAVR one (Figure 3b). K^2^_HP,SAP_(LF) decreased during POST in the SAVR group, while it remained unvaried in the TAVI one (Figure 3c). K^2^_HP,SAP_(LF) did not vary across populations within the same time point (Figure 3c). K^2^_HP,SAP_(HF) was lower in the TAVI population compared to the SAVR one in POST, while being not significantly different in PRE (Figure 3d). K^2^_HP,SAP_(HF) was stable across time points within the same population (Figure 3d).

**FIGURE 4:**
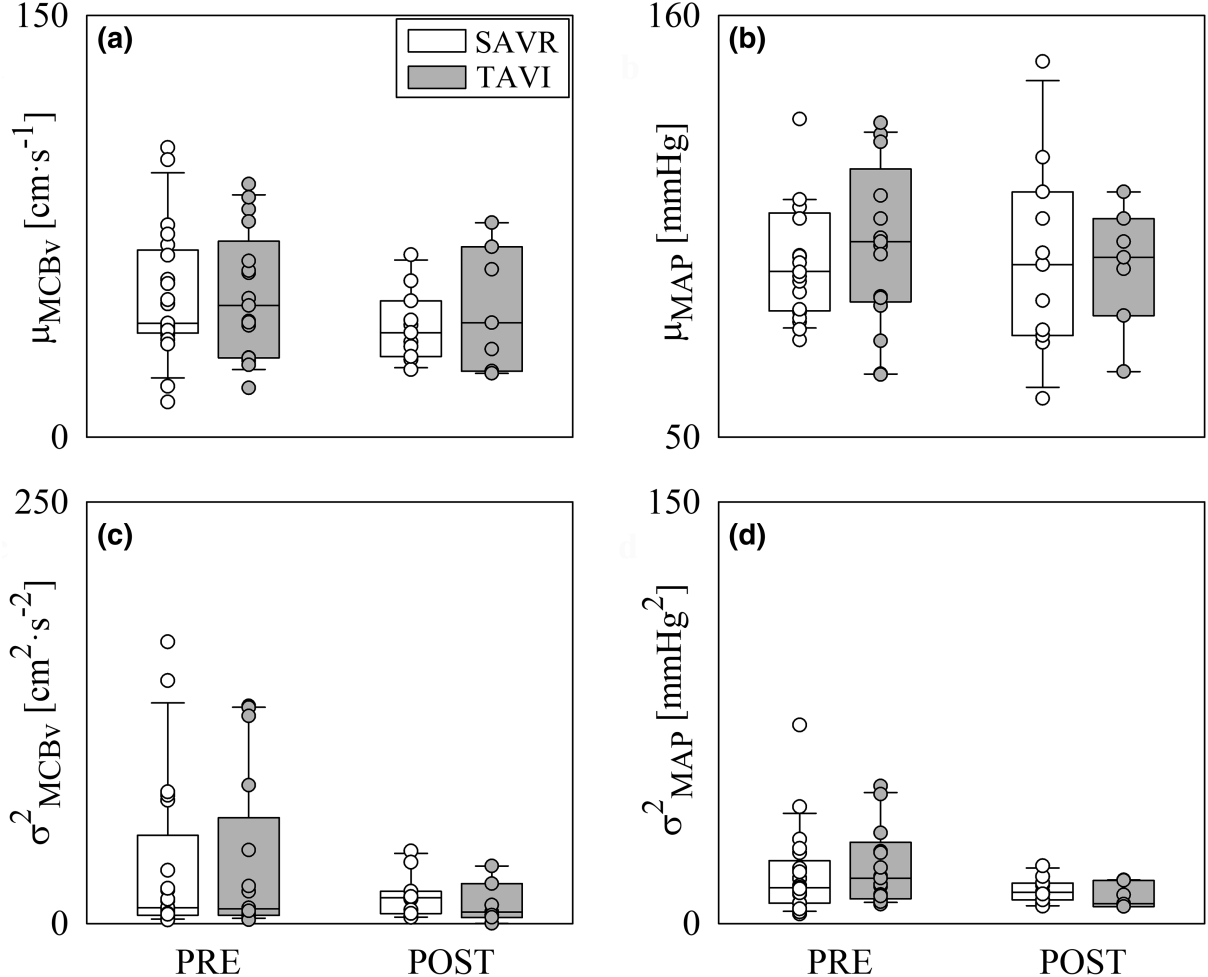
The vertical grouped box‐and‐whisker plots show μ_MCBv_ (a), μ_MAP_ (b), σ^2^_MCBv_ (c) and σ^2^_MAP_ (d) as a function of the time point of the recording (i.e. PRE and POST) in SAVR (white bars) and TAVI (gray bars) patients. Data are pooled together regardless the experimental condition (i.e., REST and STAND). The height of the box represents the distance between the first and third quartiles, with the median marked as a line, and the whiskers show the 5th and 95th percentiles. Individual values are reported as circles.

Figure 4 shows time domain CBV markers, namely μ_MCBv_ (Figure 4a), μ_MAP_ (Figure 4b), σ^2^_MCBv_ (Figure 4c) and σ^2^_MAP_ (Figure 4d). All the time domain CBV indexes did not change across either populations or time points (Figure 4a–d).

**FIGURE 5:**
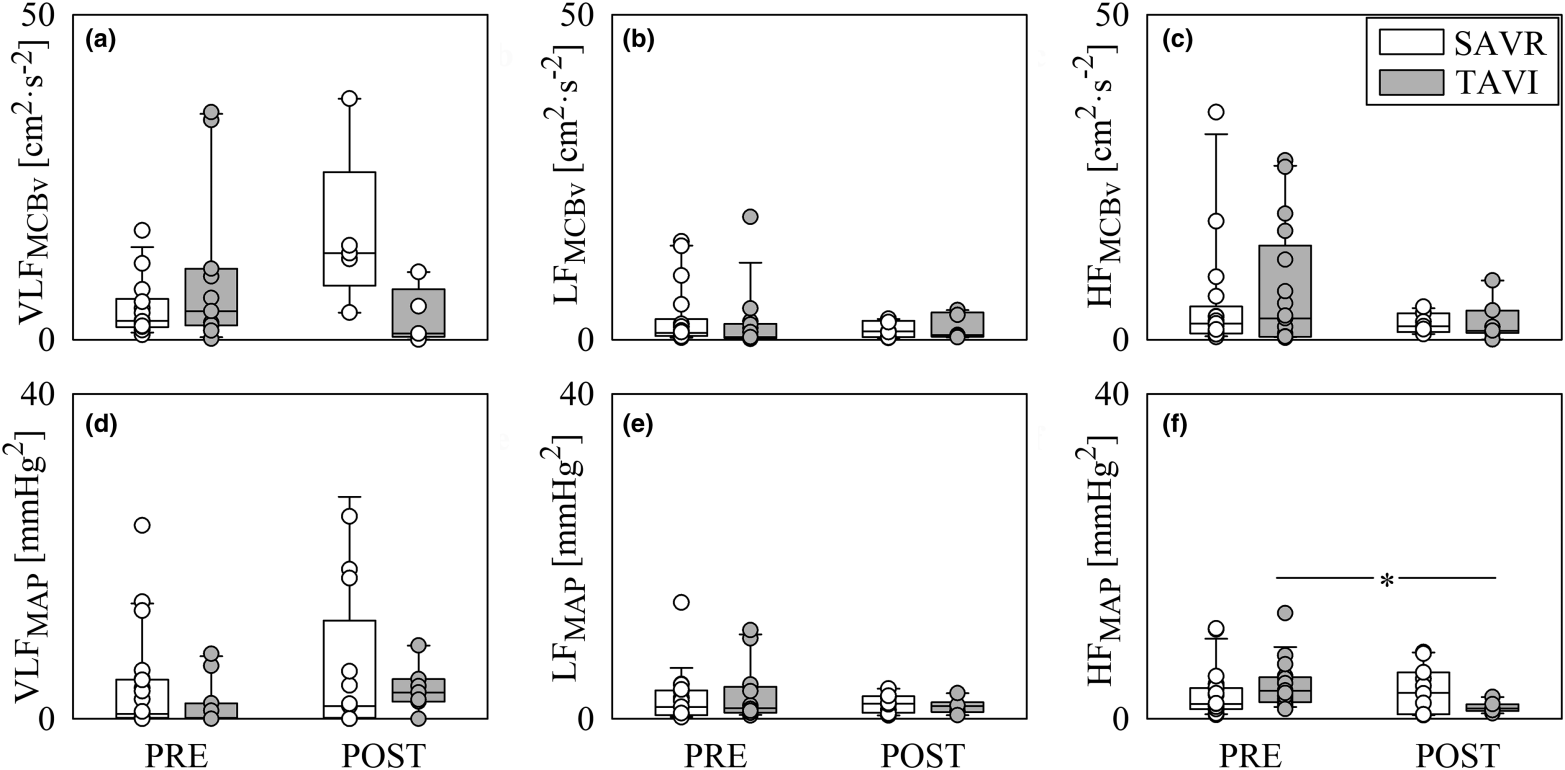
The vertical grouped box‐and‐whisker plots show VLF_MCBv_ (a), LF_MCBv_ (b), HF_MCBv_ (c), VLF_MAP_ (d), LF_MAP_ (e), and HF_MAP_ (f) as a function of the time point of the recording (i.e., PRE and POST) in SAVR (white bars) and TAVI (gray bars) patients. Data are pooled together regardless the experimental condition (i.e., REST and STAND). The height of the box represents the distance between the first and third quartiles, with the median marked as a line, and the whiskers show the 5th and 95th percentiles. Individual values are reported as circles. The symbol * indicates *p* < 0.05 between different time points within the same group.

Figure 5 shows VLF_MCBv_ (Figure 5a), LF_MCBv_ (Figure 5b), HF_MCBv_ (Figure 5c), VLF_MAP_ (Figure 5d), LF_MAP_ (Figure 5e), and HF_MAP_ (Figure 5f). Changes of univariate frequency domain CBV markers with population and time point were irrelevant with the notable exception of the decrease of the HF_MAP_ power in POST compared to PRE observed in the TAVI population (Figure 5f).

**FIGURE 6:**
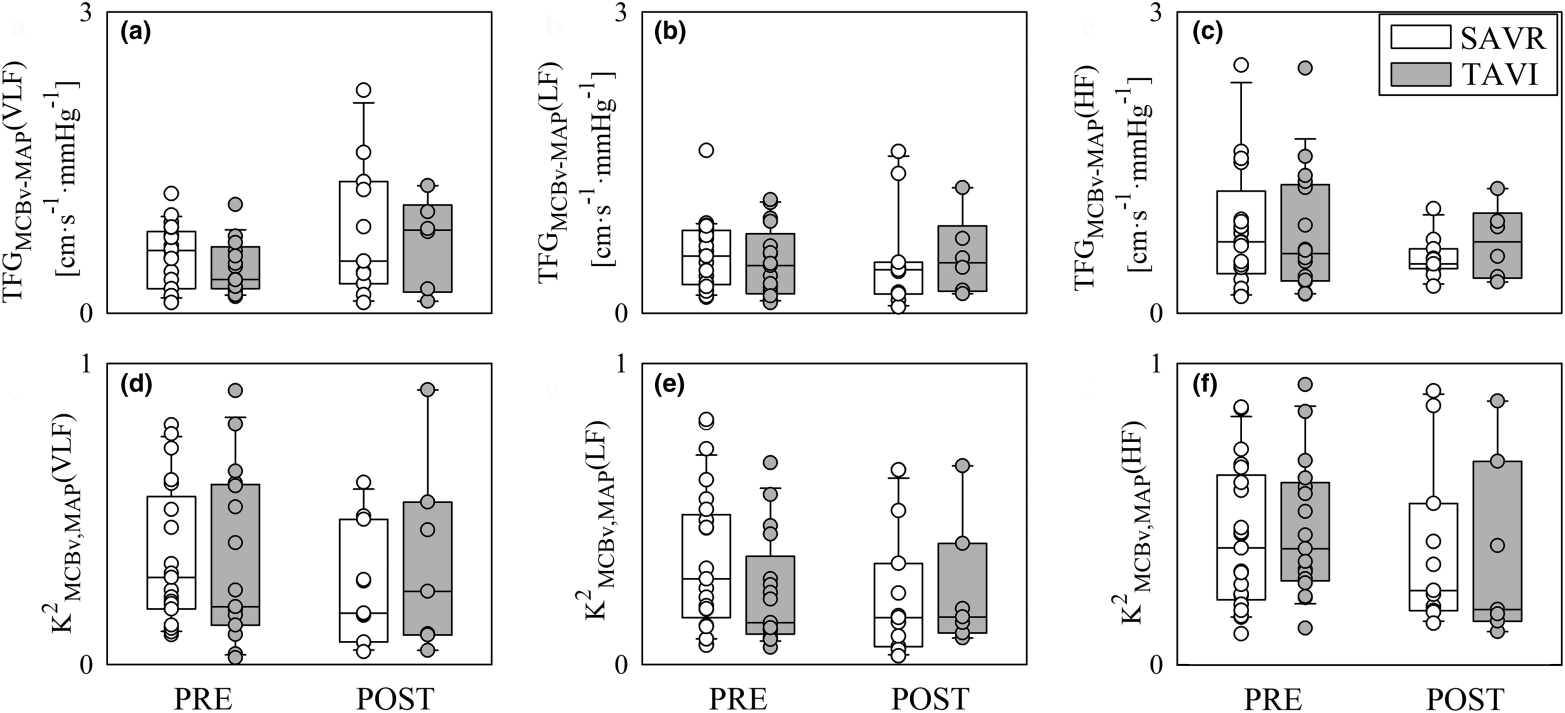
The vertical grouped box‐and‐whisker plots show TFG_MCBv‐MAP_(VLF) (a), TFG_MCBv‐MAP_(LF) (b), TFG_MCBv‐MAP_(HF) (c), K^2^_MCBv,MAP_(VLF) (d), K^2^_MCBv,MAP_(LF) (e), and K^2^_MCBv,MAP_(HF) (f) as a function of the time point of the recording (i.e., PRE and POST) in SAVR (white bars) and TAVI (gray bars) patients. Data are pooled together regardless the experimental condition (i.e., REST and STAND). The height of the box represents the distance between the first and third quartiles, with the median marked as a line, and the whiskers show the 5th and 95th percentiles. Individual values are reported as circles.

Figure 6 shows TFG_MCBv‐MAP_(VLF) (Figure 6a), TFG_MCBv‐MAP_(LF) (Figure 6b), TFG_MCBv‐MAP_(HF) (Figure 6c), K^2^_MCBv,MAP_(VLF) (Figure 6d), K^2^_MCBv,MAP_(LF) (Figure 6e), and K^2^_MCBv,MAP_(HF) (Figure 6f). The impact of the procedure on bivariate frequency domain MCBv‐MAP indexes was negligible in both groups and no differences across groups were observed within the same time point.

**FIGURE 7:**
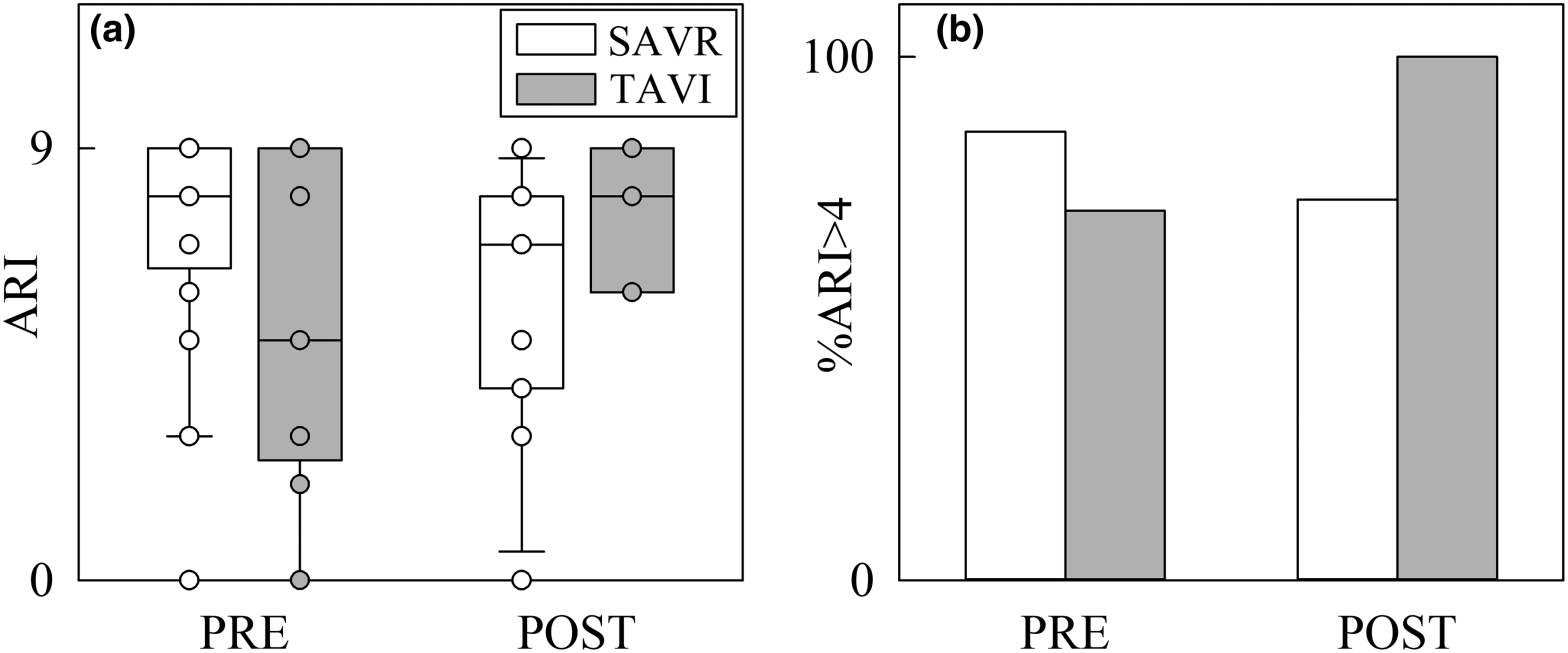
The vertical grouped box‐and‐whisker plot and vertical grouped simple bar graph show, respectively, ARI (a) and %ARI >4 (b) as a function of the time point of the recording (i.e., PRE and POST) in SAVR (white bars) and TAVI (gray bars) patients. Data are pooled together regardless the experimental condition (i.e., REST and STAND). The height of the box in (a) represents the distance between the first and third quartiles, with the median marked as a line, and the whiskers show the 5th and 95th percentiles. Individual values are reported as circles.

The vertical grouped box‐and‐whisker plot in Figure 7a shows the ARI, while the vertical grouped simple bar graph in Figure 7b depicts %ARI >4. Remarkably, in PRE ARI values were not significantly different between SAVR and TAVI groups, being %ARI >4 high in both populations. ARI and %ARI >4 were not affected by the procedure within the same population and did not vary across groups within the same time point.

## 4 DISCUSSION

The main findings of this work can be summarized as follows: (i) SAVR and TAVI groups featured a weak pre‐procedure CV control at REST; (ii) TAVI ensured a better preservation of the vagal control and baroreflex function than SAVR; (iii) CA was working in PRE in both SAVR and TAVI groups; (iv) SAVR and TAVI had no impact on the CBV control; (v) regardless of the group, CV markers were not influenced by STAND in POST; (vii) regardless of the group the impact of STAND on CBV indexes was negligible in both PRE and POST.

### 4.1 Impact of SAVR and TAVI on the CV control

We suggest that both SAVR and TAVI groups featured a pre‐procedure depression of the CV control. This indication results from the very limited values of the HP variation per unit change of SAP in the LF band and the very weak association between HP and SAP variability in the LF band observed in PRE at REST when compared to values present in literature in age‐matched healthy subjects (De Maria et al., 2019; Laitinen et al., 1998; Milan‐Mattos et al., 2018). For example, more than 70% of our subjects in PRE, either belonging to SAVR or TAVI group, had TFG_HP‐SAP_(LF) lower than the 25th percentile relevant to the oldest group of healthy individuals reported in (Milan‐Mattos et al., 2018) and this percentage increased to 100% during POST. Conversely, time domain values of HP and SAP variability are more in line with the expected values of an age‐matched healthy group (Catai et al., 2014; Laitinen et al., 2004; Milan‐Mattos et al., 2018). While some studies suggested that SAVR patients featured an important pre‐surgery impairment of the CV control (Bari et al., 2023; Porta et al., 2020; Retzlaff et al., 2009; Retzlaff et al., 2011), insufficient data are present to describe the pre‐procedure functioning of the autonomic nervous system and baroreflex control in the TAVI cohort (Compostella et al., 2015; Retzlaff et al., 2011), especially in a direct comparison with SAVR patients. Given that CV markers did not exhibit between‐group significant differences in PRE, our study suggests that autonomic control and baroreflex function in SAVR and TAVI groups might be comparably weak.

SAVR resulted in worsening the condition of CV control depression. In fact, after merging the data obtained at REST and during STAND, it was observed a post‐surgery tachycardia likely being the result of the reduced vagal drive, and a significant decrease of the magnitude of the HP fluctuations, of the BRS in the typical frequency band of baroreflex functioning in humans, namely the LF band, and of the degree of association between HP and SAP in the LF band. These results suggest a reduced vagal modulation and worsened efficiency of the cardiac arm of the baroreflex in the SAVR cohort. Although this information is not entirely new (Bari et al., 2023; Porta et al., 2020; Retzlaff et al., 2009; Retzlaff et al., 2011), it was extended in this study by considering concomitantly markers of the autonomic nervous system and baroreflex function in a context of comparison between age‐matched SAVR and TAVI patients. TAVI does not lead to a worsening of the CV control and might even induce some amelioration (Compostella et al., 2015; Dumonteil et al., 2013; Retzlaff et al., 2011). As a matter of fact, after merging the data obtained at REST and during STAND, in the TAVI group we observed that μ_HP_, σ^2^_HP_, TFG_HP‐SAP_(LF) and K^2^_HP,SAP_(LF) did not vary in POST compared to PRE, thus suggesting a more preserved CV control. The higher post‐procedure TFG_HP‐SAP_(HF) in the TAVI group compared to the SAVR one contributed to form the impression of a better post‐procedure preservation of the vagal control and baroreflex function in TAVI patients compared to the SAVR ones. Since the association of a depressed vagal activity and very limited baroreflex sensitivity with life‐threatening arrhythmias is well‐established in numerous pathological populations (Kleiger et al., 1987; La Rovere et al., 2001; Porta, Girardengo, et al., 2015; Schwartz et al., 2008), we speculate that this state of the CV control might expose SAVR patients to an additional arrhythmic risk compared to TAVI individuals. This additional CV risk might increase the rate of post‐surgery adverse events (Bari et al., 2018; Bari et al., 2019; Bauernschmitt et al., 2007; Hanada et al., 2017; Ranucci et al., 2017), thus eventually leading to lengthen the hospital stay and increase health care costs. However, despite an improvement of the baroreflex control, the σ^2^_SAP_ increased in POST in the TAVI group compared to SAVR individuals, thus suggesting a greater exposition of TAVI patients to adverse events associated with AP instabilities.

### 4.2 Impact of SAVR and TAVI on the CBV control

Since the pre‐procedure percentage of subjects with a working CA, namely with ARI >4, was 86% in the SAVR group and 73% in the TAVI one, we conclude that CBV control is operational in both groups. This observation confirmed previous findings in the SAVR group (Bari et al., 2023; Pedro et al., 2023; Porta et al., 2020; Porta, Gelpi, et al., 2022), but it was original in TAVI population. Being the group composed by elderly subjects, our data agree with the notion that aging process, despite reducing μ_MCBv_ and CBV reactivity, negligibly affects CA (Bakker et al., 2004; Lipsitz et al., 2000). In addition to confirming that ARI is preserved before the procedure in both SAVR and TAVI groups, we observed that time and frequency domain markers extracted from the MAP and MCBv variability series in SAVR and TAVI patients are not significantly different. These missing differences might indicate that SAVR and TAVI individuals feature a comparable pre‐procedure CBV control.

Impact of SAVR and TAVI on CBV control is limited. As a matter of fact, after merging the data observed at REST and during STAND, variations of ARI values and percentage of subjects with a working CA in POST compared to PRE were not significant. Spectral and cross‐spectral markers derived from MCBv and MAP variability confirmed this observation. While the negligible impact of SAVR on the CBV control has already been outlined (Bari et al., 2023; Pedro et al., 2023; Porta et al., 2020; Porta, Gelpi, et al., 2022), the finding that the same observation could be extended to TAVI is clinically relevant given that a CA impairment could be a criterion to limit the application of TAVI in patients at low and intermediate surgical risk. In addition, the minor changes of CA after SAVR and TAVI suggests that the state of the CBV control should not be considered a risk modifier of post‐procedure stroke. This information might be useful for designing more effective trials devoted to the assessment of the post‐procedure stroke risk in severe AVS patients and moves the focus from the procedure to periprocedural strategies and treatments.

### 4.3 CV control response to STAND in SAVR and TAVI populations

In response to the decrease of venous return, cardiac output, and stroke volume and consequent baroreflex unloading, STAND induces a sympathetic activation, vagal withdrawal, and baroreflex engagement in presence of smaller HP variations driven by AP changes (Cooke et al., 1999; Furlan et al., 2000; Marchi et al., 2016b; Pomeranz et al., 1985; Zaidi et al., 2000). As a result, it is expected that in a healthy population STAND induces tachycardia, a decrease of RSA and BRS, and an increase of variability of SAP and the degree of association between HP and SAP in the LF band (Cooke et al., 1999; De Maria et al., 2023; Furlan et al., 2000; Laude et al., 2004; Marchi et al., 2016a; Marchi et al., 2016b; Pomeranz et al., 1985; Porta et al., 2016). These changes were reported in elderly subjects as well, even though blunted (Bari et al., 2017; Catai et al., 2014; De Maria et al., 2019; De Maria et al., 2023; Laitinen et al., 1998; Laitinen et al., 2004). Since in PRE we found that variations of HF_HP_, LF_SAP_, TFG_HP‐SAP_(LF) and K^2^_HP,SAP_(LF) during STAND compared to REST were not significant in both SAVR and TAVI groups, we conclude that the impact of STAND on CV regulation is limited in both populations. The inability of CV control to respond to an orthostatic challenge was observed in both populations in POST as well. This observation supports once again the notion that TAVI might not be at lower risk than SAVR for AP instability, postural hypotension and adverse CV events.

### 4.4 CBV control response to STAND in SAVR and TAVI populations

In response to an orthostatic stressor, it is commonly observed in heathy subjects that μ_MCBv_ decreases and the magnitude of MCBv fluctuations increases as well as those of MAP, leading to unvaried MCBv variations per unit change of MAP in presence of a limited, but significant, increase of the degree of association between MCBv and MAP (Bari et al., 2017; Favre et al., 2020; Gelpi et al., 2022; Porta, Gelpi, et al., 2022). This increase was even more evident in old individuals (Gao et al., 2015). Similarly, ARI did not vary with an orthostatic challenge as well (Carey et al., 2001; Castro et al., 2017; Gelpi et al., 2022). Since in PRE modifications of TFG and K^2^ indexes in response to STAND were not significant in both SAVR and TAVI groups and the fraction of subjects with ARI above 4 was significant in both groups regardless of the experimental condition, we conclude that the response of the CBV control to STAND is preserved in both cohorts. Remarkably, this situation was not modified in POST. In POST only TFG_MCBv‐MAP_(HF) decreased in the TAVI group during STAND compared to REST, but this modification, suggesting an amelioration of the CBV control (Zhang et al., 1998), was not supported by any other CBV marker. While the preservation of the CBV control in the SAVR group and the limited influence of STAND were already pointed out (Bari et al., 2023; Pedro et al., 2023; Porta et al., 2020; Porta, Gelpi, et al., 2022), the notion that the same statement held in an age‐ and gender‐matched TAVI group with preserved left ventricular function might be of some value when deciding the treatment in severe AVS patients. Even the inability to separate the two groups after the application of a postural stimulus corroborates the observation that CBV markers might be useless to privilege a procedure with respect to the other.

### 4.5 Limitations of the study and future developments

The low number of subjects, especially during POST, was mainly due to post‐procedure physical and psychological debilitation of the patients. The even smaller number of subjects from which CBV control markers were computed was due to the difficulty of insonating middle cerebral arteries in elderly patients with CV diseases (Panerai et al., 2022). Since missing significant differences might be the result of the limited statistical power of the study, while some significances might be the consequence of a few individuals with abnormal values, future studies should aim at recruiting a larger population such a way that the final size of the groups in any experimental condition and time point could be more appropriate. We also acknowledge the lack of a control population limiting the power of the characterization of the pre‐procedure state of CV and CBV controls of SAVR and TAVI populations to differences between them. Several confounding factors are present given that the POST session was carried out relatively early after the procedure. Indeed, the observed values of both CV and CBV control markers in SAVR and TAVI groups could be the effect of different procedural trauma, anesthesiologic strategies, levels of inflammation, volume loading, and immobilization more than the genuine influence of the procedure on autonomic function, baroreflex and CA. Nonetheless, given the association of CV and CBV indexes with possible complications and adverse events, we deem that these markers could provide information useful to stratify the risk of TAVI and SAVR groups at the hospital discharge. A longer follow‐up would be also advisable to assure the monitoring of change of CV and CBV controls and their link with adverse events, especially with overt and silent stroke. Future analyses should be based on causal tools to explore more deeply the relationship from SAP to HP and from MAP to MCBv by accounting for causality, reflex pathways, and confounding factors such as respiration (Porta, Faes, et al., 2015; Porta, Gelpi, Bari, Cairo, De Maria, Tonon, et al., 2022; Saleem et al., 2018). Future protocols should plan to monitor the impact of arterial partial pressure of carbon dioxide at REST and during STAND, and not only just before the procedure as we did in this study.

## 5 PERSPECTIVE AND SIGNIFICANCE

We confirm the pre‐procedure weakness of the CV control in both SAVR and TAVI groups, as well as the preservation of vagal and baroreflex functions after TAVI and their worsening after SAVR. Based on these findings we suggest that SAVR group might be more at risk to develop post‐procedure episodes of cardiac arrhythmias, orthostatic hypotension and uncontrolled swings of AP than the TAVI one. Since CBV control was working in both populations before the procedures and we did not detect any significant CBV control modification after the procedure, the risk of post‐operative cerebral stroke in both the populations (Abdul‐Jawad Altisent et al., 2016; Kapadia et al., 2018) might not depend on the state of the CA, thus stressing the importance of procedure technicalities and periprocedural management in reducing neurological risk with special attention to cerebral embolic protection devices and filters as well as antiplatelet and antithrombotic therapy strategies. According to the assessment of CV and CBV regulations provided in the present study, SAVR seems to be more questionable, especially in older subjects, because the procedure is accompanied by a more depressed CV control that might expose SAVR patients to a greater post‐procedure CV risk than TAVI subjects in presence of comparable CBV risks. However, while SAVR seems to limit AP swings that cannot be controlled by baroreflex, as assessed by the SAP variance, TAVI seems to favor them, thus reducing the impact of the better preservation of baroreflex in TAVI patients compared to SAVR ones. This information might be relevant when designing more accurate clinical trials assessing the risk of adverse events and when setting health care guidelines for the treatment of AVS.

## AUTHOR CONTRIBUTIONS

V.B. and A.P: conceptualization; V.B, F.G, B.C, M.A, E.A, and M.S: data curation; V.B., F.G., and B.C.: formal analysis; M.R., and A.P: funding acquisition; V.B., and A.P. V.B., F.G., B.C., M.A., E.A., M.S. and A.P: investigation; V.B., F.G., B.C., and A.P: methodology; A.P: project administration; V.B., M.R., and A.P: resources; V.B., F.G., B.C., and A.P: software; A.P.: supervision; V.B., and A.P.: validation; V.B., and A.P: visualization; V.B., and A.P: writing, original draft; V.B., F.G., B.C., M.A., E.A., M.S., B.D.M., E.G.B., V.F., E.C., C.D.V., F.B., M.R., and A.P: writing, review and editing.

## FUNDING INFORMATION

No financial or otherwise, are declared by the authors.

## CONFLICT OF INTEREST STATEMENT

No conflicts of interest.

## Data Availability

Data will be made available upon reasonable request.
